# Adrenergic/Cholinergic Immunomodulation in the Rat Model—*In Vivo* Veritas?

**DOI:** 10.1155/1998/30686

**Published:** 1998

**Authors:** I. Rinner, P. Felsner, P. M. Liebmann, D. Hofer, A. Wölfler, A. Globerson, K. Schauenstein

**Affiliations:** 1 lnstitute of General and Experimental Pathology University of Graz Austria; 2 Department of Immunology Weizmann Institute of Science Rehovot Israel

**Keywords:** Neuroimmunomodulation, catecholamines, acetylcholine, lymphocytes, thymic epithelial cells, apoptosis, choline-acetyl transferase, dopamine-beta-hydroxylase

## Abstract

For several years, our group has been studying the *in vivo* role of adrenergic and cholinergic mechanisms in the immune-neuroendocrine dialogue in the rat model. The main results of these studies can be summarized as follows: (1) exogenous or endogenous catecholamines suppress PBL functions through alpha-2-receptor-mediated mechanisms, lymphocytes of the spleen are resistant to adrenergic *in vivo* stimulation, (2) direct or indirect cholinergic treatment leads to enhanced *ex vivo* functions of splenic and thymic lymphocytes leaving PBL unaffected, (3) cholinergic pathways play a critical role in the “talking back” of the immune system to the brain, (4) acetylcholine inhibits apoptosis of thymocytes possibly via direct effects on thymic epithelial cells, and may thereby influence T-cell maturation, (5) lymphocytes of the various immunological compartments were found to be equipped with the key enzymes for the synthesis of both acetylcholine and norepinephrine, and to secrete these neurotransmitters in culture supernatants


*Developmental Immunology*, 1998, Vol. 6, pp. 245–252
Reprints available directly from the publisher
Photocopying permitted by license only

© 1998 OPA (Overseas Publishers Association)
N.V. Published by license under the
Harwood Academic Publishers imprint,
part of the Gordon and Breach Publishing Group
Printed in Malaysia

### Adrenergic/Cholinergic Immunomodulation in the Rat Model—*In Vivo* Veritas?

I. RINNER[a*], P. FELSNER[a], P.M. LIEBMANN[a], D. HOFER[a], A. WÖLFLER[a], A. GLOBERSON[b] and K. SCHAUENSTEIN[a]

[a]*Institute of General and Experimental Pathology, University of Graz, Austria;* [b]*Department of Immunology, Weizmann Institute of Science, Rehovot, Israel*

(*Received 1 October 1996; In final form 10 February 1997*)

For several years, our group has been studying the *in vivo* role of adrenergic and cholinergic mechanisms in the immune-neuroendocrine dialogue in the rat model. The main results of these studies can be summarized as follows: (1) exogenous or endogenous catecholamines suppress PBL functions through alpha-2-receptor-mediated mechanisms, lymphocytes of the spleen are resistant to adrenergic *in vivo* stimulation, (2) direct or indirect cholinergic treatment leads to enhanced *ex vivo* functions of splenic and thymic lymphocytes leaving PBL unaffected, (3) cholinergic pathways play a critical role in the "talking back" of the immune system to the brain, (4) acetylcholine inhibits apoptosis of thymocytes possibly via direct effects on thymic epithelial cells, and may thereby influence T-cell maturation, (5) lymphocytes of the various immunological compartments were found to be equipped with the key enzymes for the synthesis of both acetylcholine and norepinephrine, and to secrete these neurotransmitters in culture supernatants

*Keywords:* Neuroimmunomodulation, catecholamines, acetylcholine, lymphocytes, thymic epithelial cells, apoptosis, choline-acetyl transferase, dopamine-beta-hydroxylase

### INTRODUCTION

Investigations in the past decades have unraveled close mutual relationships between the neuroendocrine and the immune systems (Cotman et al., 1987; Chelmicka-Schorr and Arnason, 1990). Central and peripheral lymphoid organs are innervated by the sympathetic and parasympathetic systems with nerve terminals forming "synapsislike" contacts to the immune cells (Bulloch and Moore, 1981; Felten et al., 1987). Inversely, T lymphocytes were found to regularly invade the central nervous system at random (Hickey et al., 1991). A great variety of common receptors, surface proteins, and mediators are expressed at high levels in both the neuroendocrine and the immune systems providing information

*Corresponding author.

exchange within as well as between the systems. Adrenergic, muscarinic, and nicotinic cholinergic, and many peptidergic receptors have been identified on lymphocytes (Plaut, 1987). Activation of these receptors can exert immunosuppressive or enhancing effects, depending on the respective signal molecule, the type of immune cell concerned (Blalock, 1989), and the different microenvironments of lymphoid tissues (Felsner et al., 1992; Rinner and Schauenstein, 1991; Rinner et al., 1992). The generation of neurohormones, neuropeptides, and neurotransmitters by thymocytes and peripheral lymphocytes has been documented in several species (Smith et al., 1990; Rinner and Schauenstein, 1993), and, on the other hand, neural and endocrine tissues are known to express lymphokines and monokines as well as high densities of their membrane receptors (Velasco et al., 1991; Cunningham and Souza, 1993). Functionally, cytokines influence endocrine and central nervous functions, including activation of the hypothalamic-pituitary adrenal and hypothalamic-godanal axes, induction of fever or slow wave sleep, and signaling to the structures of the "limbic system" (Haas and Schauenstein, 1997).

Our group is interested in defining the role of the autonomous nervous system within the dialogue between the central nervous system and the immune system. In the rat model, we could show that the *in vivo* treatment with adrenergic and cholinergic agonists had pronounced and partly opposite effects on *ex vivo* functions of lymphocytes (Rinner and Schauenstein, 1991; Felsner et al., 1992, 1995), and that the cholinergic system particularly takes part in the signaling from the peripheral immune system to the brain (Rinner and Schauenstein, 1991).

### ALPHA-ADRENERGIC SUPPRESSION OF PBL PROLIFERATIVE RESPONSE IN THE RAT MODEL

The literature data on adrenergic immunoregulation are conflicting; both suppression and enhancement have been reported as a consequence of alpha- or beta-adrenergic stimulation (Hadden et al., 1970; Pearlman, 1971; De Pelchin and Letesson, 1981; Felten et al., 1987; Felten and Felten, 1994; Madden et al., 1995). These differences are probably due to differences in the experimental approach, such as, for example, *in vivo* versus *in vitro* studies, species differences, and epiphenomena due to "handling stress" of laboratory animals (Rinner et. al., 1992).

In our own studies, we tried to define the *in vivo* effects of chronically enhanced levels of peripheral catecholamines on *ex vivo* functions of lymphocytes of rats. The experimental model consists of s.c. implantation of retard tablets that provide a controlled release of adrenergic agonists or antagonists during 24 hr. The results obtained so far are summarized as follows (Figure 1): 24 hr of administration of adrenaline (or noradrenaline) did not or only marginally change the proliferative response of peripheral blood lymphocytes (PBL) to Concanavalin A (Con A). Concomitant administration of the beta-receptor blocker propranolol, but not of an alpha blocker, caused a pronounced and significant decrease of the mitogenic response of PBL (Felsner et al., 1992). This inhibition was shown to be mediated via alpha-2-adrenergic receptors and it could be likewise induced by administration of an indirect sympathomimetic drug (tyramine) in combination with beta blockade (Felsner et al., 1995). The adrenergic immunosuppression was not due to general stress phenomena mediated by glucocorticoids, or shifts in total T cells ($CD3^+$) or T-cell subsets ($CD4^+CD8^+$). Surprisingly, in our hands, this effect was strictly confined to PBL; spleen cells were consistently resistant to direct or indirect adrenergic treatment, even though this organ is known to be strongly innervated with sympathetic terminals. A similar divergent sensitivity of spleen cells and PBL was previously observed during immobilization stress, which induced strong inhibition of the mitogenic response of PBL, but increased the reactivity of splenic cells (Rinner et al., 1992). It is possible that adrenergic receptors of splenic lymphocytes, which are in close contact to adrenergic fibers, are desensitized due to high basal catecholamine levels.

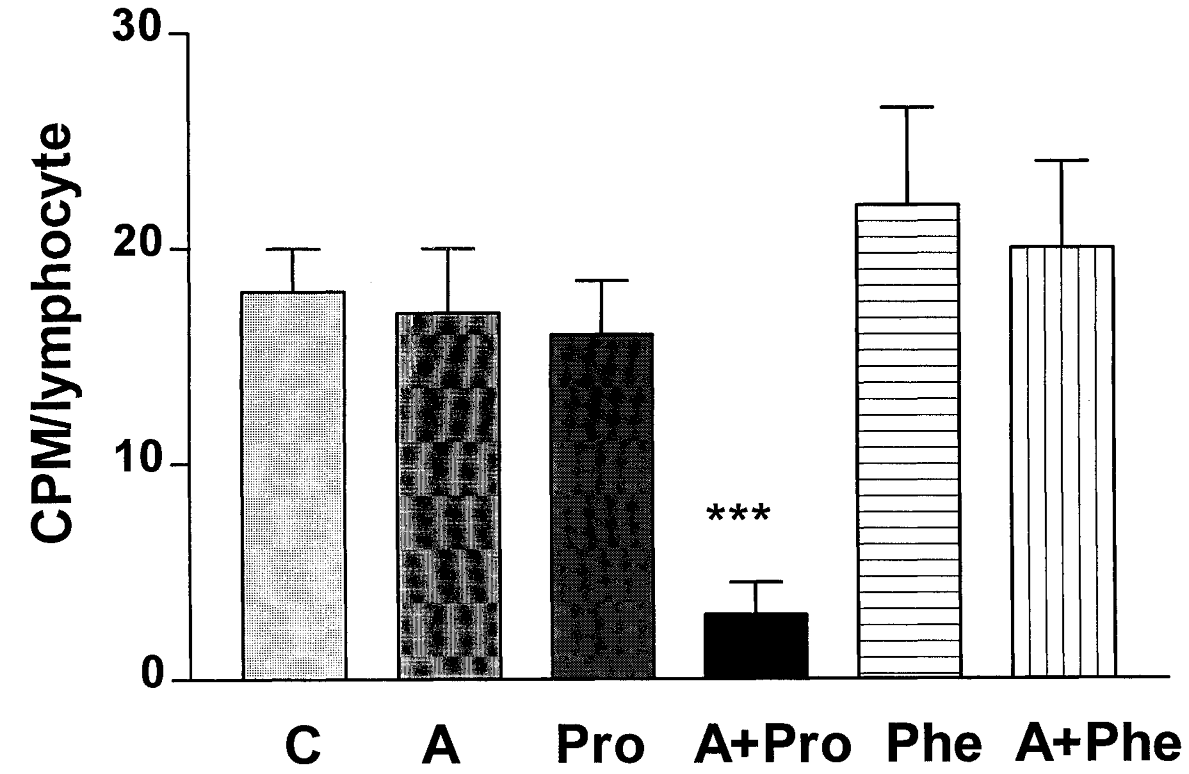

FIGURE 1 Concanavalin A response of peripheral blood lymphocytes of rats in whole-blood stimulation. *In vivo* treatment for 24 hr with constant release of pellets containing placebo (C); adrenaline (A); 1.31 ± 0.04 μg/min propranolol (Pro); 3.13 ± 0.31 μg/min of adrenaline + propranolol (A + Pro); phentolamine (Phe); and 1.32 ± 0.02 μg/min of adrenaline + phentolamine (A + Phe). Rats were implanted subcutaneously with retard tablets containing adrenaline, either alone or in combination with a blocker containing a retard tablet. Placebo tablets consisted of the tablet matrix without a drug. After 24 hr, the animals were killed, blood was collected, and the spleens were excised. Mitogen stimulation was determined in the whole blood and in spleen-cell suspensions after 3 days. Data represent the means ± SEM of maximal 3HTdR uptake related to the lymphocyte number in culture (CPM/lymphocyte). $N = 6$ for each group.*** $p < 0.01$ significantly different from controls according to Student's $t$-test.

Our *in vivo* results are in contrast to reports of beta-adrenoceptor-mediated immunosuppression by catecholamines *in vitro* (Plaut, 1987) and *in vivo* (Chelmicka-Schorr et al., 1993). They also suggest that, besides enhanced catecholamine blood levels, adrenenergic immunosuppression requires a bias in the alpha/beta adrenergic receptor balance. Recently, we obtained evidence that endogenous melatonin confers a strong protection to lymphocytes against the suppressive effect of alpha-adrenergic stress. Beta-adrenergic blockers inhibit the pineal release of melatonin, and by virtue of this enforce the suppressive effects of catecholamines (Liebmann et al., 1996).

### CHOLINERGIC ENHANCEMENT OF *EX VIVO* FUNCTIONS OF SPLENIC AND THYMIC LYMPHOCYTES

Since lymphocytes express muscarinic and nicotinic cholinergic receptors and the developing T cells in the thymus are not only in contact with the sympathetic, but also with the parasympathetic cholinergic system, we tested the effects of cholinergic treatment on lymphocyte functions. Sprague Dawley rats were implanted s.c. with retard tablets continuously releasing the muscarinic cholinergic blocker atropine or the inhibitor of acetylcholine esterase physostigmine. After 5 days, the animals were sacrificed and the *ex*

vivo Con A response of lymphocytes was determined. Figure 2 shows the effects for PBL, and splenic and thymic lymphocytes. Whereas PBL are unaffected by any treatment, the mitogenic stimulation of spleen cells is strongly suppressed by muscarinic receptor blockade, and the response of thymocytes is significantly increased by inhibition of acetylcholine degradation by physostigmine. As with the adrenergic effects, we are not able to explain the selective effects of atropine and physostigmine on cells from different immunological compartments. Further studies are needed to solve this puzzle.

### CHOLINERGIC PATHWAYS ARE INVOLVED IN AFFERENT SIGNALING OF THE IMMUNE SYSTEM TO THE BRAIN

Besedovsky et al. (1975) were first to describe a regulatory feedback loop between the immune system and the central nervous system. Cytokines released from activated immune cells stimulate via specific receptors the hypothalamic-pituitary-adrenal axis leading to a transient increase in glucocorticoids in the blood. This mechanism seems to be important for the control of "forbidden clones," as defects in this immuno-neuroendocrine feedback were shown in several species to be associated with spontaneous or experimentally induced autoimmune diseases (Schauenstein et al., 1987; Sternberg et al., 1990). We investigated the effect of the chronic administration of physostigmine and/or atropine on the corticosterone response in rats 4 days after i.p. immunization with sheep red blood cells (Figure 3). Physostigmine abrogated the rise in corticosterone; atropine had no effect, but antagonized the suppression induced by physostigmine. These data provided first evidence that cholinergic mechanisms are involved in the feedback regulation between the immune system and brain. Further studies are presently underway to determine if and to what degree alterations in the endogenous cholinergic tonus may predispose an animal to autoimmune reactions.

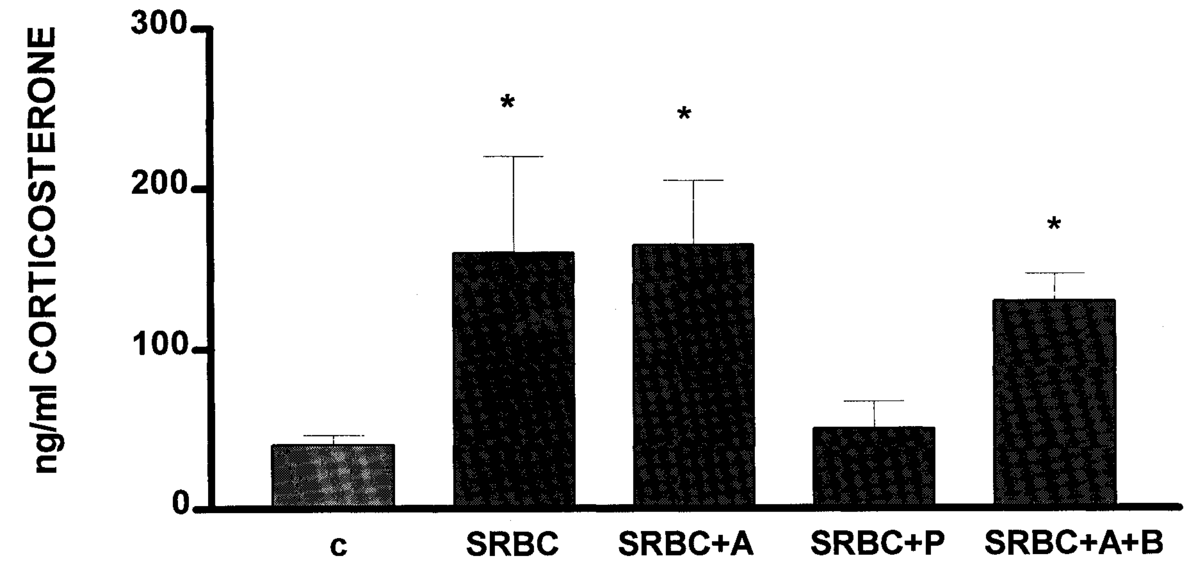

FIGURE 2 Effect of 5 days' treatment with constant release pellets containing placebo, atropine (1.2 mg/day), or physostigmine (0.012 mg/day) on concanavalin-A-stimulated 3H-TdR uptake of lymphocytes from the peripheral blood (WBS, whole-blood stimulation), the spleen, and the thymus. Rats were implanted subcutaneously with retard tablets containing atropine or physostigmine. Placebo tablets consisted of the tablet matrix without a drug. After 5 days, the animals were killed, blood was collected, and the spleens and thymuses were excised. Mitogen stimulation was determined in the whole blood and in spleen- and thymus-cell suspensions after 3 days. ;Each group represents the mean ± SEM of seven rats. Significance of the difference from placebo-treated animals: * $p < 0.05$ to Student's *t*-test.

### ACETYLCHOLINE INHIBITS APOPTOSIS OF THYMOCYTES VIA A DIRECT EFFECT ON THYMIC EPITHELIUM AND MAY THEREBY INFLUENCE T-CELL MATURATION

During T-cell maturation, nonreactive and autoreactive thymocytes are not positively selected or eliminated by negative selection. The nonselected and the negatively selected cells die by the induction of apoptosis, the programmed cell death. This selection is primarily based on cellular interactions between the developing cells and the thymic epithelium, but soluble factors produced by the epithelial cells can modulate this process. In a coculture system, consisting of nontransformed thymic epithelial cell (TEC) lines (Hirokawa et al., 1986) and fetal thymic lobes, we investigated the effect of this coculture on thymocyte apoptosis and development (Rinner et al., 1994, 1996). Coculture of fetal thymic lobes with a cortical (TEC 1.4) and a medullary (TEC 2.3) TEC line induced a decrease in cell number and an increase of apoptotic cells in the thymus lobes (Table I), which was significant with cortical TEC 1.4 cells only. This effect was found to be restricted to thymic lymphocytes, and to be mediated by a > 30-kD factor in the supernatant of TEC lines. Addition of the cholinergic drug carbachol attenuated the apoptosis induction by TEC 1.4 cells, but did not influence the apoptosis in lobes without TECs, nor in presence of the medullary TEC 2.3 line, suggesting that the primary targets of the cholinergic effect are indeed the cells of the cortical epithelial cell line. The attenuation could be antagonized by d-tubocurarine, a nicotinic receptor blocking agent (not shown). Coculture of thymic

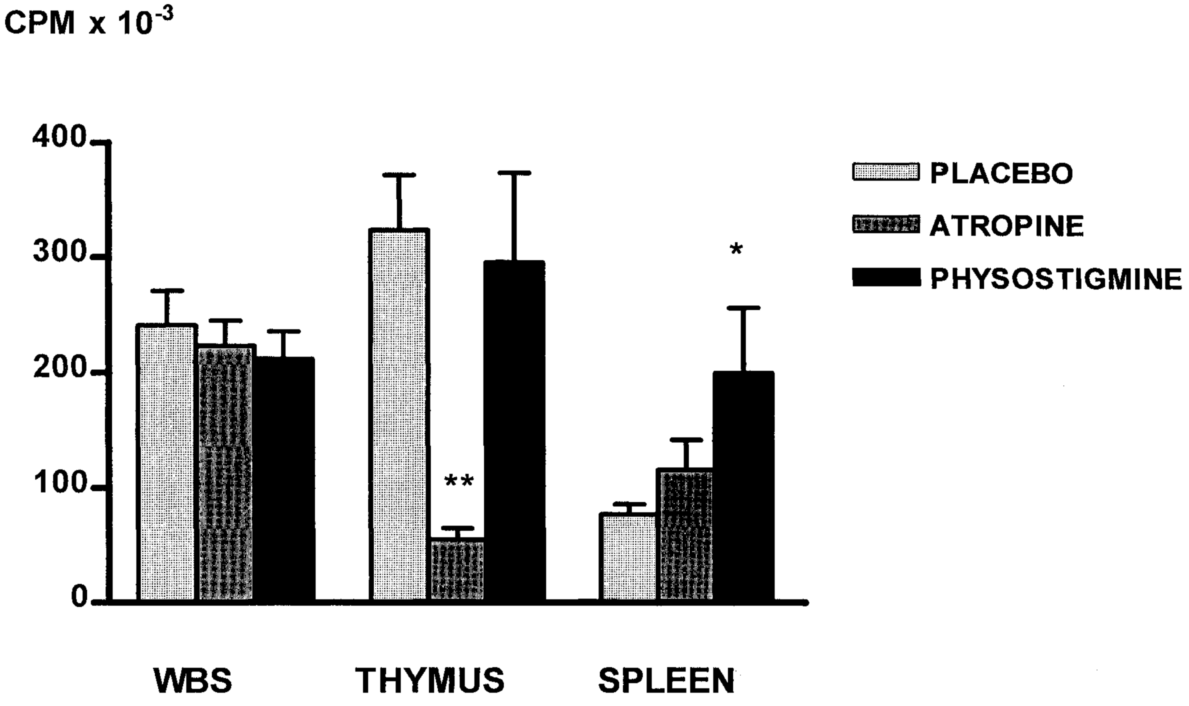

FIGURE 3 Effect of chronic cholinergic stimulation by physostigmine administration on corticosterone elevation 4 days after immunization with sheep red blood cells. Control: no immunization; SRBC: $10^9$ sheep red blood cells i.p.; SRBC+A: administration of atropine (3 mg/kg) on the day of immunization and the following day; SRBC+P: administration of physostigmine (0.3 mg/kg) on the day of immunization and the following day; SRBC+A+P: administration of physostigmine and atropine on the day of immunization and the following day. The animals were killed, corticosterone was extracted from the diluted plasma, and the concentration of corticosterone was determined by RIA. Each group represents the means ± SEM of eight rats. Statistical difference was determined using ANOVA, followed by Dunnet's *t*-test.

TABLE 1 Effect of *In Vitro* Treatment with Carbachol on the Percentage of Apoptotic Cells in Murine Fetal Thymus Cocultured with Cortical (TEC 1.4) of Medullary (TEC 2.3) Epithelial Cells

| | No TEC | TEC 1.4 | TEC 2.3 |
|---|---|---|---|
| Control | 100.0 ± 1.8 | 307.3 ± 1.2[c] | 165.5 ± 19.4 |
| $10^{-8}$M carbachol | 95.8 ± 16.4 | 257.6 ± 28.4[c] | 112.1 ± 26.1 |
| $10^{-7}$ M carbachol | 101.2 ± 14.5 | 207.9 ± 9.1[a,b] | 105.5 ± 24.8 |
| $10^{-7}$ M carbachol | 115.2 ± 10.3 | 267.3 ± 12.2[c] | 98.2 ± 12.1 |

Tec cells were seeded on Nucleopore filters resting on gelatine sponges. Fetal thymus lobes (day 15 after gestation) were placed on top of each layer or control sponges without TEC. The cocultures were daily treated with the respective drug. Data are presented as percentage of controls. Results are means ± SEM of three independent experiments.[a]Significantly different ($p < 0.01$) from the respective control without drug.

[b]Significantly different ($p < 0.01$) from cultures without TEC according to ANOVA and Student-Newaman-Keuls post hoc analysis.

[c]Significantly different ($p < 0.001$) from cultures without TEC according to ANOVA and Student-Newaman-Keuls post hoc analysis.

lobes with TEC 1.4, but not with TEC 2.3, also decreased the percentage of CD4/CD8 double-positive cells and increased the percentage of CD4/CD8 double-negative cells (not shown). Taken together, these data indicate that soluble factor(s) produced by cortical epithelial cells are able to regulate apoptosis and differentiation of thymocytes, and that cholinergic signals can modulate this effect.

### LYMPHOCYTES EXPRESS CHOLINE-ACETYL TRANSFERASE AND DOPAMINE-BETA-HYDROXYLASE, AND SECRETE ACETYLCHOLINE AND NOREPINEPHRINE IN CULTURE SUPERNATANTS

Besides the classical immune cytokines, that is, the interleukins, interferons, and other factors, all mediating pleiotropic effects on many different tissues, lymphocytes were found to also produce mediators previously thought to exclusively belong to the neuroendocrine system, such as products of the POMC gene and hormones of the anterior pituitary gland (Blalock, 1989). We have tested the hypothesis that immune cells are also able to produce neurotransmitters of the autonomous nervous system. Using the methods of Fonnum (1975) and Nagatsu and Udenfriend (1972), we could detect the activities of the key enzymes of acetylcholine and norepinephrine synthesis, that is, choline-acetyltransferase (ChAT) and dopamine-$\beta$-hydroxylase, in lymphocyte homogenates (Table II). Activities of both enzymes could be detected in thymic, splenic, and peripheral blood lymphocytes to a similar extent. Furthermore, very recently, the ChAT message could be detected by RT-PCR methods in thymus cells, splenocytes, and PBL (Rinner et al., to be published).

The concentration of acetylcholine in lymphocytes and in culture supernatants was measured by a sensitive RIA (Kawashima et al., 1980). Figure 4(A) shows that thymocytes, spleen cells, and PBL contain similar amounts of this neurotransmitter. Stimulation with PHA significantly increased both the cellular content and the release of acetylcholine into the

TABLE 2 Choline Acetyltransferase (ChAT) and Dopamine-$\beta$-Hydroxylase (DbH) Activity in Homogenates of Rat Thymic Cells, Spleen Cells, and Peripheral Blood Lymphocytes (PBL) (pmol $\times$ mg$^{-1}$ $\times$ min$^{-1}$).

| | ChAT | DbH |
|---|---|---|
| Thymus cells | 34.5 ± 2.8 | 204.4 ± 24.3 |
| Spleen cells | 22.9 ± 3.3 | 128.6 ± 18.1 |
| PBL | 13.8 ± 1.6 | 163.0 ± 13.9 |
| Fibroblasts | N.D | N.D. |

Cultured rat fibroblasts were used as negative control for the enzyme activities. 1 $\times$ $10^7$ cells were homogenized by sonication, centrifuged, and the enzyme activity was determined in aliquots of the supernatant using the method of Fonnum (1975) and Nagatsu and Udenfriend (1972), respectively. Results are means ± SEM. $N = 3$. N.D.: not detectable.

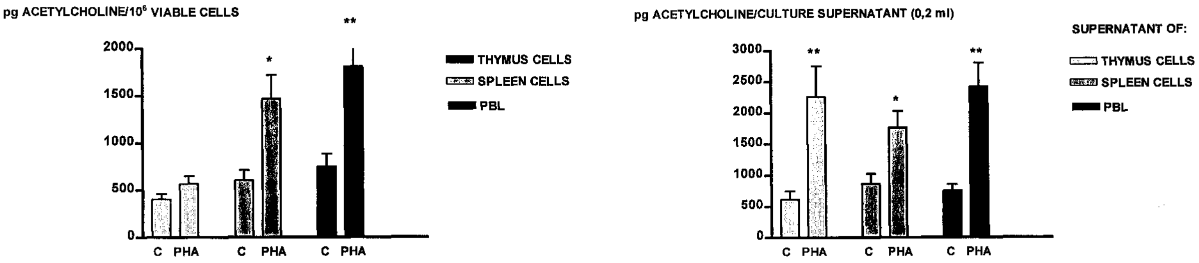

FIGURE 4 Acetylcholine content in (A) lymphatic cells and in (B) the culture medium of the cells from thymus, spleen, ande peripheral blood. 1 × $10^6$ cells were homogenized by sonication. Protein was denaturated by perchloric acid and the neutralized supernatant was used for the acetylcholine RIA according to Kawashima et al. (1980). Results are means ± SEM. $N = 6$.

culture medium; see Figure 4(B). Intra- and extracellular catecholamines were determined by the HPLC technique of Aigner and McManus (1985) using electrochemical detection. Norepinephrine could be detected in cellular extracts and culture supernatants of spleen and PBL (5. 3 ± 0.39 and 1. 06 ± 0. 11 pg/$10^6$ cells; N = 6), but not of thymus cells. Mitogenic stimulation did not change this pattern (not shown).

More studies are needed to explore the secretory mechanism(s) by which lymphocytes release neurotransmitters, and to examine the physiological relevance of these phenomena to immunoregulation *in vivo*. Nevertheless, the fact that lymphocytes do not only react to but also are able to produce adrenergic and cholinergic neurotransmitters suggests close and mutual relationships to the autonomous nervous system, and may add to the concept of "the immune system as mobile brain."

### Acknowledgements

The experiments reviewed herein were supported by the Austrian Science Foundation (projects 7509, 7038, 9925, 10422, and 1130) and by the "Jubiläumsfonds der Osterreichischen Nationalbank" (projects 3556 and 4349).

### References

Aigner H. and McManus J. (1985). Bestimmung der Katecholamine mit neuem Detektor and Chemikalien-Kit. Labor-Medizin **2**: 110-112.

Besedovsky H., Sorkin E., Keller M. and Müller J. (1975). Changes in blood hormone levels during the immune response. Proc. Soc. Exp. Biol. Med. **150**: 466-470.

Blalock J.E. (1989). A molecular basis for bidirectional communication between the immune and neuroendocrine systems. Physiol. Rev. **69**: 1-27.

Bulloch K. and Moore R.Y. (1981). Innervation of the thymus gland by brain stem and spinal cord in mouse and rat. Am. J. Anat. **162**: 157-166.

Chelmicka-Schorr E. and Arnason B.W. (1990). Nervous system-immune system interactions. In Immunologic Mechanisms in Neurologic and Psychiatric Disease, Waksman B.H., Ed. (New York: Raven Press), pp. 67-90.

Chelmicka-Schorr E., Wollmann R.L., Kwasniewski M.N., Kim D.H. and Dupont B.L. (1993). The $\beta 2$-adrenergic agonist terbutaline suppresses acute passive transfer experimental autoimmune myasthenia gravis (EAMG). Int. J. Immunopharmacol. **15**: 19-24.

Cotman C.W., Brinton R.E., Galaburda A., McEwen B. and Schneider D.M. (1987). The Neuro-Immune-Endocrine Connection (New York: Raven Press).

Cunningham E.T. and Souza E.B. (1993). Interleukin-1 receptors in the brain and endocrine tissues. Immunol. Today **14**: 171-176.

De Pelchin A. and Letesson J.J. (1981). Adrenaline influence on the immune response. I. Accelerating of suppressor effects according the time of application. Immunol. Lett. **3**: 199-205.

Felsner P., Hofer D., Rinner I., Mangge H., Gruber M., Korsatko W. and Schauenstein K. (1992). Continuous in vivo treatment with catecholamines suppresses in vitro reactivity of rat peripheral blood T-lymphocytes via alpha-mediated mechanisms. J. Neuroimmunol **37**: 47-57.

Felsner P., Hofer D., Rinner I., Porta S., Korsatko W. and Schauenstein K. (1995). Adrenergic suppression of peripheral blood T cell reactivity in the rat is due to activation of peripheral $\alpha_2$-receptors. J. Neuroimmunol. **57**: 27-34.

Felten D.L., Felten S.Y., Bellinger D.L., Carlson S.L., Ackerman K.D., Madden K.S., Olschowski J.A. and Livnat S. (1987). Noradrenergic sympathetic neural interactions with the immune system: Structure and function. Immunol. Rev. **100**: 225-260.

Felten S.Y. and Felten D.L. (1994). Neural-immune interactions. Prog. Brain Res. **100**: 157-162.

Fonnum F.A. (1975). Rapid radiochemical method for the determination of choline acetyltransferase. J. Neurochem. **24**: 407-409.

Haas S. and Schauenstein K. (1997). Neuroimmunomodulation via limbic structures. The neuroanatomy of psychoimmunology. Prog. Neurobiol. **51**: 195-222.

Hadden J.W., Hadden E.M. and Middelton E. (1970). Lymphocyte blast transformation. I. Demonstration of adrenergic receptors in human peripheral lymphocytes. Cell Immunol. **1**: 583-595.

Hickey W.F., Hsu B.L. and Kimura H. (1991). T-Lymphocyte entry into the central nervous system. J. Neurosci. Res. **28**: 254-260.

Hirokawa K., Utsuyama M., Moriizumi E. and Handa S. (1986). Analysis of the thymic microenvionment by monoclonal antibodies with special reference to the nurse cell. Thymus **112**: 449-455.

Kawashima K., Ishikawa H. and Mochizuki M. (1980). Radioimmunoassay for acetylcholine in the rat brain. J. Pharmacol. Meth. **3**: 115-123.

Liebmann P.M., Hofer D., Felsner P., Wölfler A. and Schauenstein K. (1996). Beta blockade enhances adrenergic immunosuppression in rats via inhibition of melatonin release. J. Neuroimmunol. **67**: 137-142.

Madden K.S., Felten S.Y., Felten D.L. and Bellinger D.L. (1995). Sympathetic nervous system-immune system interactions in young and old Fischer 244 rats. Ann. N.Y. Acad. Sci. **771**: 523-534.

Nagatsu T. and Udenfriend S. (1972). Photometric assay of dopamine-$\beta$-hydroxylase activity in human blood. Clin. Chem. **18**: 980-983.

Pearlman D.S. (1971). The effect of adrenergic agents on the antibody response in vitro. J. Allergy Clin. Immunol. **47**: 109-110.

Plaut M. (1987). Lymphocyte hormone receptors. Annu. Rev. Immunol. **5**: 261-269.

Rinner I., Eren R., Skreiner E., Kukulansky T., Kasai M., Hirokawa K., Globerson A. and Schauenstein K. (1996). Thymocyte-directed enhancement of apoptosis via soluble factor(s) derived from a cortical and a medullary thymic epithelial cell line. Cell Tis. Res. **284**: 327-330.

Rinner I., Kukulansky T., Felsner P., Skreiner E., Globerson A., Kasai M., Hirokawa K., Korsatko W. and Schauenstein K. (1994). Cholinergic stimulation modulates apoptosis and differentiation of murine thymocytes via a nicotinic effect on thymic epithelium. Biochem. Biophys. Res. Comm. **203**: 1057-1062.

Rinner I. and Schauenstein K. (1991). The parasympthetic nervous system takes part in the immuno-neuroendocrine dialogue. J. Neuroimmunol. **34**: 165-172.

Rinner I. and Schauenstein K. (1993). Detection of choline-acetyltransferase activity in lymphocytes. J. Neurosci. Res. **35**: 188-191.

Rinner I., Schauenstein K., Mangge H., Porta S. and Kvetnansky R. (1992). Opposite effects of mild and severe stress on in vitro activation of rat peripheral blood lymphocytes. Brain Behav. Immun. **6**: 130-140.

Schauenstein K., Fässler R., Dietrich H., Schwarz S., Krömer G. and Wick G. (1987). Disturbed immune-neuroendocrine communication in autoimmune disease. Lack of corticosterone response to immune signals in Obese Strain chickens with spontaneous autoimmune thyreoiditis. J. Immunol. **139**: 1830-1833.

Smith E.M., Galin F.S., LeBoef R.D., Coppenhaver D.H., Harbour D.V. and Blalock J.E. (1990). Nucleotide and amino acid sequence of lymphocyte derived corticotropin: Endotoxin induction of a truncated peptide. Proc. Natl. Acad. Sci. USA **87**: 1057-1060.

Sternberg E., Wilder R.L., Gold P.W. and Chrousos D.P. (1990). A defect in the central component of the immune system-hypothalamic-pituitary-adrenal axis feedback loop is associated with susceptibility to experimental arthritis and other inflammatory diseases. Ann. N.Y. Acad. Sci. **594**: 289-292.

Velasco S., Tarlow M., Olson K., Shay J.W., McCracken G.H. and Nielsen P.D. (1991). Temperature-dependent modulation of lipopolysaccharide-induced interleukin-1 beta and tumor necrosis factor-alpha expression in cultured human astroglial cells by dexamethason and indomethacin. J. Clin. Invest. **87**: 1674-1680.

